# Evaluation of microvascular endothelial function in patients with infective endocarditis using laser speckle contrast imaging and skin video-capillaroscopy: research proposal of a case control prospective study

**DOI:** 10.1186/s13104-017-2660-3

**Published:** 2017-07-28

**Authors:** Amanda Barcelos, Cristiane Lamas, Eduardo Tibiriça

**Affiliations:** 1 National Institute of Cardiology, Ministry of Health, Rio de Janeiro, Brazil; 2 National Institute of Infectious Diseases Evandro Chagas, Oswaldo Cruz Institute, Rio de Janeiro, Brazil; 3 Unigranrio University, Rio de Janeiro, Brazil; 4 Laboratory of Cardiovascular Investigation, Oswaldo Cruz Institute, Av. Brasil 4365, Rio de Janeiro, 21045-900 Brazil

**Keywords:** Infective endocarditis, Microcirculation, Microvascular dysfunction, Speckle contrast imaging, Skin video-capillaroscopy

## Abstract

**Objective:**

Infective endocarditis is a severe condition with high in-hospital and 5-year mortality. There is increasing incidence of infective endocarditis, which may be related to healthcare and changes in prophylaxis recommendations regarding oral procedures. Few studies have evaluated the microcirculation in patients with infective endocarditis, and so far, none have utilized laser-based technology or evaluated functional capillary density. The aim of the study is to evaluate the changes in the systemic microvascular bed of patients with both acute and subacute endocarditis. This is a cohort study that will include adult patients with confirmed active infective endocarditis according to the modified Duke criteria who were admitted to our center for treatment. A control group of sex- and age-matched healthy volunteers will be included. Functional capillary density, which is defined as the number of spontaneously perfused capillaries per square millimeter of skin, will be assessed by video-microscopy with an epi-illuminated fiber optic microscope. Capillary recruitment will be evaluated using post-occlusive reactive hyperemia. Microvascular flow will be evaluated in the forearm using a laser speckle contrast imaging system for the noninvasive and continuous measurement of cutaneous microvascular perfusion changes. Laser speckle contrast imaging will be used in combination with skin iontophoresis of acetylcholine, an endothelium-dependent vasodilator, or sodium nitroprusside (endothelium independent) to test microvascular reactivity.

**Results:**

The present study will contribute to the investigation of microcirculatory changes in infective endocarditis and possibly lead to an earlier diagnosis of the condition and/or determination of its severity and complications.

*Trial registration* ClinicalTrials.gov ID: NCT02940340.

## Introduction

Infective endocarditis (IE) is a disease with a high mortality rate that can potentially lead to severe complications. Its clinical presentation is dynamic and variable; it is dependent on patient age, the presence of comorbidities (especially underlying valvular heart disease), the causative microorganism and the presence of complications. In recent years, there has been a change in its epidemiological profile, especially in developed countries. Although younger patients with rheumatic valve disease were the predominant population affected by IE, in the last two decades, older individuals and healthcare-associated infections (including those related to intracardiac devices, valve prosthesis and hemodialysis) and a staphylococcal etiology have become more frequent [1, 2]. However, in developing countries, patients with rheumatic valve disease account for a third of all IE cases, and the *viridans* group streptococci is the causative agent in a third of all IE cases [3].

Infective endocarditis is a systemic disease involving the vascular system and is often accompanied by bacteremia or fungaemia. Therefore, it results in a septic state. In addition, acute heart failure or acute-on-chronic heart failure commonly complicates left-sided IE due to valve destruction, which leads to acute valvular regurgitation. Indeed, the most common indication for valve replacement surgery is severe heart dysfunction not amenable to pharmacologic intervention [1–3]. Considering the severity and increasing incidence of IE, it is important to better understand the pathophysiology of the endocarditis syndrome. The use of noninvasive techniques in the early diagnosis of IE and its complications may prove useful. In fact, the evaluation of systemic microvascular reactivity has proven to be very helpful in the investigation of the pathophysiology of cardiovascular and metabolic disorders [4, 5] as well as sepsis [6].

Abnormalities in the microcirculation have been demonstrated through microvascular rarefaction to be present in diseases such as arterial hypertension, diabetes, obesity and metabolic syndrome [5, 7–11]. Moreover, impaired systemic microvascular function, characterized primarily by capillary rarefaction in the skin, has been demonstrated in individuals with increased coronary artery disease risk [12, 13].

The importance of vascular endothelial function in cardiovascular medicine has also been demonstrated using microRNAs (miRs), non-coding RNAs that can regulate gene expression via translational repression and/or post-transcriptional degradation [14]. One of the examples is miR-126, which plays a fundamental role in homeostasis and in the modulation of vascular development. Endothelial function can also be regulated by miRs commonly expressed in endothelial cells. One of the best examples of this is miR-223, a cholesterol homeostasis regulator [14].

Laser speckle contrast imaging, which is used to assess skin microvascular reactivity, allows for innovative and reproducible noninvasive evaluation of tissue flow with high real-time spatial resolution in patients with cardiometabolic diseases [15, 16] and critically ill patients [17, 18]. Moreover, cutaneous microvascular reactivity has been correlated to microvascular function in different vascular beds, both in intensity and regarding the underlying mechanisms [19].

Although IE is the prototype of a septic condition that results in acute heart dysfunction, only one study has addressed microcirculation in infective endocarditis [20], and thus far, none have utilized laser speckle contrast imaging or functional skin video-capillaroscopy. Studies have shown microvascular changes in sepsis, in which abnormalities are found in early phases even before the deterioration of hemodynamic parameters [21]. These studies show the relationship of microcirculatory changes with organ failure and mortality, which are independent of the systemic hemodynamic variables [21]. It is probable that in acute staphylococcal endocarditis, findings similar to those in the studied sepsis models may be encountered [17, 22].

The goal of this study is to assess microvascular density and reactivity in patients with acute and subacute endocarditis by laser speckle contrast imaging and skin video-capillaroscopy.

## Main text

### Methods

#### Study design and place

This is a cohort study that will include patients with a confirmed diagnosis of infective endocarditis who were admitted to the National Institute of Cardiology (NIC) at the Ministry of Health in Rio de Janeiro, Brazil. The NIC is a national reference center for the treatment and research of cardiovascular diseases. Its staff is composed of cardiologists, cardiothoracic surgeons, infectious diseases specialists, specialized nursing staff, physiotherapists and pharmacists as well as technical staff. The investigative resources include echocardiography, computed tomography, magnetic resonance imaging and scintigraphy. The NIC has outpatient units, four intensive care units and operating theatres where approximately 1300 cardiac surgeries are performed yearly.

#### Study participants and recruitment

Patients will be recruited consecutively and prospectively from the endocarditis cohort. The eligibility criteria are as follows: (1) confirmed IE according to the modified Duke criteria [23]; (2) inpatient treatment at the NIC; (3) clinical stability as evaluated by the investigator; and (4) age ≥ 18 years. The exclusion criterion is a confirmed previous diagnosis of *diabetes mellitus*.

#### Study variables

The variables to be included are as follows: demographic data (sex, age and ethnicity), previous medical history (comorbidities, previous valvular diseases and cardiac interventions), medications in use, data regarding the episode of IE (date of onset, causative pathogen, affected valve or structure, echocardiographic data, local and systemic embolic and non-embolic complications, antibiotic and surgical treatment) and laboratory data (CRP, ESR and creatinine levels).

#### Intervention

The evaluation of microvascular endothelial function in patients with infective endocarditis will be performed using laser speckle contrast imaging and skin video-capillaroscopy. These results will be compared to those previously obtained from age- and sex-matched healthy volunteers [13]. The systemic microvascular data obtained from this group of healthy volunteers will be used as reference microcirculatory values of individuals free of systemic diseases. None of the healthy volunteers presented with arterial hypertension, diabetes, dyslipidemia or any other systemic pathology.

#### Evaluation of microcirculatory reactivity

The microcirculatory tests will be performed in the morning between 8 a.m. and 12 p.m. in an undisturbed, quiet room with a defined stable temperature (23 ± 1 °C) following a 20-min rest period in the supine position. The room temperature will be monitored and adjusted if necessary using air conditioning. The acclimatization period will last until each patient’s skin temperature stabilizes [24]. We have previously demonstrated that following 15–20 min of acclimatization, the skin temperature stabilizes at approximately 29 °C [8].

#### Evaluation of skin microvascular flow and reactivity

Microvascular reactivity will be evaluated using a laser speckle contrast imaging (LSCI) system with a laser wavelength of 785 nm (PeriCam PSI system, Perimed, Järfälla, Sweden), as previously described [25]. LSCI will be used in combination with the iontophoresis of acetylcholine (ACh) or sodium nitroprusside (SNP) for the non-invasive, continuous measurement of cutaneous microvascular perfusion changes in arbitrary perfusion units (APUs). The images will be analyzed using the manufacturer’s software (PIMSoft, Perimed, Järfälla, Sweden). One skin site on the ventral surface of the forearm will be randomly chosen for the recordings. Hair, broken skin, areas of skin pigmentation and visible veins will be avoided, and a single drug-delivery electrode will be installed using adhesive discs (LI 611, Perimed, Järfälla, Sweden). A vacuum cushion (AB Germa, Kristianstad, Sweden) will be used to reduce the recording artifacts generated by arm movements. The iontophoresis of ACh 2% w/v or SNP 2% w/v (Sigma Chemical CO, USA) will be performed using a micropharmacology system (PF 751 PeriIont USB Power Supply, Perimed, Sweden) with increasing anodal currents of 30, 60, 90, 120, 150 and 180 μA for 10-s intervals that are spaced 1 min apart, and the total charges will be 0.3, 0.6, 0.9, 1.2, 1.5 and 1.8 mC. The dispersive electrode will be attached approximately 15 cm away from the iontophoresis chamber. The results of the pharmacological tests will be expressed both as peak values (representing the maximal vasodilation observed following the highest dose of ACh or SNP) and as the area under the curve of vasodilation. The measurements of skin blood flow will be divided by mean arterial pressure values to provide the cutaneous vascular conductance (CVC) in APUs/mmHg.

Microvascular reactivity will also be evaluated using a physiological test known as post-occlusive reactive hyperemia (PORH). During the PORH test, arterial occlusion will be achieved with supra-systolic pressure (50 mmHg above the systolic arterial pressure) using a sphygmomanometer for 3 min. Following the release of pressure, maximum flux will be measured.

#### Capillaroscopy by intra-vital video-microscopy

Capillary density, i.e., the number of perfused capillaries per mm^2^ of skin area, will be assessed by high-resolution intra-vital color microscopy (Moritex, Cambridge, UK), as previously described [8]. Capillaroscopy will be performed using a video microscopy system with an epi-illuminated fiber optic microscope containing a 100-W mercury vapor lamp light source and an M200 objective with a final magnification of 200×. The dorsum of the non-dominant middle phalanx will be used for image acquisition, which will occur while the patient is seated comfortably in a constant temperature environment (23 ± 1 °C). Images will be acquired and saved for subsequent offline analysis using a semi-automatic integrated system (Microvision Instruments, Evry, France). Mean capillary density will be calculated as the arithmetic mean of the number of visible (i.e., spontaneously perfused) capillaries in three contiguous microscopic fields of 1 mm^2^ each. A blood pressure cuff will then be applied to the patient’s arm and inflated to supra-systolic pressure (50 mmHg above the measured systolic arterial pressure) to interrupt blood flow completely for three minutes. After cuff release, images will again be acquired and recorded for 60–90 s, during which a maximal hyperemic response is expected to occur [26–28]. All images will be analyzed by two independent observers who will have no knowledge of the subject’s group assignment (control subjects or patients).

#### Sample size calculations

The prospective power analysis was based on data from previous studies from our group using intravital video-microscopy. This analysis indicated that a sample size of 16 subjects per group would have 80% power at the 5% significance level to detect a difference of 7-capillaries/mm^2^ (the standard deviation) during PORH between groups. The calculations were made using classical power calculations with the formula.

$$ n = f(\alpha ,\beta ) \cdot \frac{{2s^{2} }}{{\delta^{2} }} $$

where *α* is the significance level, *β* is the power of the test, *f* (α, β) is a value calculated from *α* and *β* (in this case 7.9), *δ* is the difference in means that we should be able to detect, and *s* is the standard deviation determined in previous studies.

#### Statistical analysis

The results will be presented as the mean ± SD. For values that do not follow a Gaussian distribution, the medians (25th–75th percentiles) will be presented (Shapiro–Wilk normality test). The results will be analyzed using either two-tailed unpaired Student’s *t*-tests or repeated measures ANOVAs when appropriate. The independent (unpaired) *t* test will be used because we will compare two unrelated groups, in which the participants (healthy individuals or patients with IE) in each group are different. P values <0.05 will be considered statistically significant. The statistical package to be used for the statistical analyses is Prism version 6.0 (GraphPad Software Inc. La Jolla, CA, USA).

Clinical and laboratory data will be shown descriptively. The correlations between the intervention study results (capillary density and microvascular reactivity) and features of the disease, such as the number of days of presentation, presence of embolic complications and etiological agent, will be determined using Pearson’s test if the data are found to be of normal distribution (parametric). If the distribution is not normal (non-parametric), Spearman’s test will be used for the analysis.

### Discussion

Infective endocarditis remains a disease with high mortality (approximately 15–20% among patients who undergo surgery) and morbidity despite improvements in diagnosis and timely surgical intervention [1, 2]. It is associated with persistent bacteremia or fungaemia and consequent sepsis. Studies evaluating the microcirculation in patients with endocarditis are scarce in the literature. Only one French study, in which nailfold capillary microscopy was used to study twenty-six patients with IE, showed significant correlations of the number of capillary abnormalities with systemic involvement and immunological disturbances [20]. However, the authors concluded that due to the lack of specificity, nailfold capillary microscopy could not be regarded as a useful tool for the diagnosis of infective endocarditis. Another important issue to consider is the relatively frequent occurrence of severe valvular regurgitation leading to acute heart failure in left-sided IE, which may further compromise microcirculation and tissue perfusion, possibly modifying the response of the microvascular endothelium to sepsis.

Several studies have demonstrated the central role of microcirculation in the delivery of nutrients and oxygen to tissue cells and, therefore, in determining adequate organ perfusion [29, 30]. In sepsis and septic shock, macro and microcirculatory disturbances both contribute to the development of organ failure. Macro and microcirculatory mismatch in septic patients may lead to inappropriate treatment measures and higher mortality [31]. Microcirculation and its effects are a focus of present and future research that aim to improve diagnostic and treatment strategies. Moreover, it has recently been demonstrated that treatment at an early stage of microvascular dysfunction may be most effective for delaying or reversing the disease processes, thus improving the outcome and survival of patients at risk for pulmonary vascular disease [32]. The authors also suggested that this knowledge may be applied toward microvascular dysfunction observed in the skin [32].

In sepsis, microvascular blood flow is disturbed by endothelial dysfunction, which leads to a reduction in flow or no flow at all to some capillary beds, while other beds have above normal flow, even surpassing tissue metabolic demands. This microvascular perfusion heterogeneity results in tissue hypoxia. Endothelial dysfunction in sepsis also results in an inflammatory response, which together with tissue hypoxia, leads to organ failure and mortality [31, 33]. Intravenous fluids and vasoactive agents are important measures in the treatment of sepsis. Intravenous fluids may improve microvascular perfusion, increasing the proportion of perfused capillaries and decreasing flow heterogeneity. However, systemic circulatory changes may be relatively independent of changes in the microcirculation [33, 34].

Our study proposes to evaluate the baseline cutaneous functional capillary density and capillary recruitment (capillary reserve) in patients with IE using PORH. Capillary rarefaction and heterogeneous capillary flow may be shown [18]. In addition to using a laser speckle contrast imaging system, whether microvascular reactivity is normal may be demonstrated with acetylcholine, an endothelium-dependent vasodilator, and sodium nitroprusside, an endothelium-independent vasodilator drug. The results of these studies may be helpful in providing information on the systemic involvement of microcirculation in infective endocarditis.

## Limitations and strengths of the study

- The restricted number of participants due to limitations regarding the inclusion of critically ill patients with a diagnosis of infective endocarditis.
- The heterogeneity of patients presenting with IE, which is due to several differences, including underlying diseases, such as chronic valvular disease (present or not) and chronic renal failure (present or not and hemodialysis dependent or not); etiologic agents, which may be more aggressive, such as *Staphylococcus aureus*, or more indolent, such as *viridans* group streptococci; the type of structure involved, such as the mitral or aortic valves and the tricuspid valve (associated with devices or not); the type of treatment received by the patient (surgical vs. non-surgical); and the duration of treatment at the time of the study protocol.
- The main strength of the present study is the assessment of the usefulness of laser-based noninvasive techniques in the evaluation of systemic microcirculation and its role in the early diagnosis of infective endocarditis and, possibly, its complications.
